# Short periods of high temperature during meiosis prevent normal meiotic progression and reduce grain number in hexaploid wheat (*Triticum aestivum* L.)

**DOI:** 10.1007/s00122-017-2925-1

**Published:** 2017-05-26

**Authors:** Tracie Draeger, Graham Moore

**Affiliations:** Crop Genetics Department, John Innes Centre, Norwich Research Park, Norwich, NR4 7UH UK

## Abstract

***Key message*:**

**Exposure of wheat to high temperatures during male meiosis prevents normal meiotic progression and reduces grain number. We define a temperature-sensitive period and link heat tolerance to chromosome 5D.**

**Abstract:**

This study assesses the effects of heat on meiotic progression and grain number in hexaploid wheat (*Triticum aestivum* L. var. Chinese Spring), defines a heat-sensitive stage and evaluates the role of chromosome 5D in heat tolerance. Plants were exposed to high temperatures (30 or 35 °C) in a controlled environment room for 20-h periods during meiosis and the premeiotic interphase just prior to meiosis. Examination of pollen mother cells (PMCs) from immature anthers immediately before and after heat treatment enabled precise identification of the developmental phases being exposed to heat. A temperature-sensitive period was defined, lasting from premeiotic interphase to late leptotene, during which heat can prevent PMCs from progressing through meiosis. PMCs exposed to 35 °C were less likely to progress than those exposed to 30 °C. Grain number per spike was reduced at 30 °C, and reduced even further at 35 °C. Chinese Spring nullisomic 5D-tetrasomic 5B (N5DT5B) plants, which lack chromosome 5D, were more susceptible to heat during premeiosis–leptotene than Chinese Spring plants with the normal (euploid) chromosome complement. The proportion of plants with PMCs progressing through meiosis after heat treatment was lower for N5DT5B plants than for euploids, but the difference was not significant. However, following exposure to 30 °C, in euploid plants grain number was reduced (though not significantly), whereas in N5DT5B plants the reduction was highly significant. After exposure to 35 °C, the reduction in grain number was highly significant for both genotypes. Implications of these findings for the breeding of thermotolerant wheat are discussed.

## Introduction

Wheat is the most widely grown cereal crop in the world, and of the three major cereal food crops (wheat, rice and maize), it is the most sensitive to temperature increases (Tripathy et al. 2009). Global temperatures are predicted to rise throughout the 21st Century (Intergovernmental Panel on Climate Change 2014), and it has been estimated that for each °C of temperature increase, global wheat production will decrease by 6% (Asseng et al. 2015), and that an increase in temperature of only 4 °C could result in major risks to food security (Porter et al. 2014).

Wheat is more sensitive to high temperature during its reproductive phase than when it is in its vegetative phase (Fischer and Maurer 1976). The optimum temperature range for wheat growth is generally considered to be around 17–23 °C over the course of an entire growing season (Porter and Gawith 1999), and temperatures higher than this during wheat’s floral development can result in a reduction in grain yield (Fischer and Maurer 1976; Fischer 1985; Wardlaw et al. 1989). Meiosis is particularly sensitive to heat stress. Saini and Aspinall (1982), for example, observed a substantial lowering in grain yield when wheat plants were exposed to 30 °C for 1–3 days between the beginning of meiosis and anthesis, and Alghabari et al. (2014) found that meiosis is particularly susceptible to heat if water is withheld. The latter study relied on the assumption that meiosis occurs when wheat is at the ‘booting’ stage of development, when the developing spike within the flag leaf sheath becomes visibly enlarged. However, assigning meiosis to specific growth stages by assessing the external morphology of a plant with the naked eye has been reported to be unreliable (Barber et al. 2015).

Greater accuracy in determining the start and end points of meiosis and of its various phases can be achieved by dissection of the floral organs and examination of the PMCs. Using this method, the duration of meiosis and of each of its stages has been elucidated in the wheat variety Chinese Spring (Bennett et al. 1971, 1973). Meiosis is immediately preceded by premeiotic interphase, which, at 20 °C, takes about 48 h, and can be divided into three cytologically distinct stages: stage 1 lasting around 18 h and stages 2 and 3 lasting around 15 h each (Bennett and Smith 1972). Stages 1 and 2 together are largely equivalent to G1 in the somatic cell cycle, and it is thought that S-phase, during which DNA replication takes place, probably begins during stage 2 and ends during late stage 3 (Bennett et al. 1979). It is unclear whether there is a short G2 after DNA synthesis.

In Chinese Spring, at 20 °C, meiosis takes approximately 24 h. It begins with prophase, during which homologous chromosomes pair and recombine. Prophase can be further subdivided, by the cytological appearance of chromosomes in the PMCs, into leptotene, zygotene, pachytene, diplotene and diakinesis. This is followed by metaphase I then two cell divisions resulting in four haploid daughter cells, also known as microspores or pollen cells. Leptotene has been reported to last for 10.4 h at 20 °C, zygotene for 3.4 h and the rest of meiosis from pachytene onwards for 10.2 h (Bennett et al. 1971). However, high temperatures can shorten the duration of meiosis in wheat and other grasses. Bennett et al. (1972) observed that in Chinese Spring, meiosis takes only 18 h at 25 °C, and in the diploid grass *Dasypyrum villosum* (L.) P. Candargy, meiosis was reported to last for 29 h at 20 °C, 21 h at 28 °C and only 17 h at 35 °C (Stefani and Colonna 1996).

In addition to altering the duration of meiosis, high temperatures can induce a variety of meiotic aberrations including irregular chromosome segregation (laggards), chromosome bridges, micronuclei (Rezaei et al. 2010) and changes in chiasma frequency (Dowrick 1957). In 1972 (a), Bayliss and Riley studied the effects of both high and low temperatures on chiasma frequency in Chinese Spring euploid plants and in plants lacking chromosome 5D (Chinese Spring nullisomic 5D-tetrasomic 5B [N5DT5B] plants). They found that, at temperatures above 30 °C, the chromosomes become too ‘sticky’ to allow accurate scoring, but in the few cells where chiasma could be observed at 35 °C, chiasma frequency appeared to be lower in the plants lacking chromosome 5D than in euploid Chinese Spring plants, indicating that plants lacking chromosome 5D might be more sensitive to high temperature than those with a normal chromosome complement.

At low temperatures, chiasma frequency is easier to score. At 15 °C, N5DT5B plants have a greatly reduced chiasma frequency when compared with Chinese Spring euploids, and they show pronounced chromosome pairing failure at 12 °C, leading to complete male sterility (Riley 1966; Hayter and Riley 1967). Plants tetrasomic for chromosome 5B (20 disomic chromosomes and four 5B chromosomes) have normal pairing at high or low temperatures, so the chromosome pairing failure does not appear to be a result of the extra dosage of chromosome 5B (Riley et al. 1966). It was, therefore, inferred that chromosome 5D stabilises chromosome pairing at low temperatures and probably at high temperatures too. Low chiasma frequencies in N5DT5B plants are due to the chromosomes failing to pair during zygotene (Bayliss and Riley 1972a), but the temperature-sensitive period occurs earlier, during stage 2 of premeiotic interphase, prior to DNA synthesis (Bayliss and Riley 1972b). Chromosome pairing in N5DT5A plants is near normal at low temperatures, suggesting that a double dose of chromosome 5A compensates for the deficiency of chromosome 5D, but in plants nullisomic for 5A, synapsis is apparently normal at 12 °C (Riley et al. 1966). This indicates that chromosome 5A has a weak stabilising effect on chromosome pairing at low temperatures.

More recently, it has been postulated that there are several genes on wheat group 5 chromosomes that have a stabilising effect on meiotic chromosome pairing or recombination at low temperatures, including two on chromosome 5D. These have been named *Ltp1* (low temperature pairing) and *Ltr* (low temperature recombination) (Queiroz et al. 1991), but, as yet, none of these group 5 genes have been mapped. In fact, relatively little is known of the role of individual genes controlling heat tolerance in wheat (Mullarkey and Jones 2000), and there are surprisingly few studies of the effects of heat on meiosis, despite its obvious importance. This lack of research could be partly due to the difficulty of pinpointing when meiosis is occurring without using time-consuming invasive techniques.

In this study, Chinese Spring euploid and N5DT5B plants were exposed to short pulses (20 h) of high temperatures (30 and 35 °C) during specific stages of meiosis and of the premeiotic interphase just prior to meiosis. To ensure plants were being exposed at a known stage of meiosis or premeiosis, an anther sampling technique was used (Bennett and Smith 1972; Bennett et al. 1971, 1974), which involves excision of anthers from immature spikes immediately before and after heat treatment, followed by microscopic examination of PMCs undergoing male meiosis. The aims of the study were to assess the effects of high temperatures on meiotic cell progression and grain number, to identify the most heat-sensitive stages of meiosis and to investigate the role of chromosome 5D in heat tolerance.

## Materials and methods

### Plant materials

The plants used in this study were two wheat (*Triticum aestivum* L., 2*n* = 6*x* = 42) genotypes of the variety Chinese Spring: one with the standard euploid form, AABBDD, and the other its nullisomic 5D-tetrasomic 5B (N5DT5B) genotype, which lacks chromosome 5D. Plants were grown in pots in a controlled environment room (CER) at 20 °C (day) and 15 °C (night) with a 16-h photoperiod (lights on between 10:00 and 02:00) and 70% humidity, until development of the main shoot or tiller to be sampled had progressed to between Zadoks growth stage 39 and stage 43 (Zadoks et al. 1974; Tottman 1987). At stage 39, the flag leaf ligule is just visible and the PMCs in the most developed anthers are normally at premeiotic interphase. At stage 41, the flag leaf sheath is extending and meiosis is in progress. At stage 43 the stem is just visibly swollen by the developing spike at early booting, and PMCs in the most well developed anthers are progressing through the latter stages of meiosis. During these stages, when the immature spike was still tightly enclosed within the leaf sheaths, a single anther was excised, to accurately determine the stage of premeiosis or meiosis of the PMCs prior to high temperature treatment.

To estimate the developmental stage of each main shoot or tiller more precisely before anther excision, two external features were measured: the first was the length of flag leaf sheath showing above the junction of the blade and sheath of the penultimate (boot) leaf (the distance between the flag and boot leaf collars), and the second was the approximate length of the undissected spike. Spike length was assessed by gently feeling for the large, easily detectable node immediately below the spike, and, just above this node, locating the peduncle (the internode immediately below the developing spike). Spike length was measured as the distance between the thickest part of the peduncle and the top of the spike. Chinese Spring is awnless, but this method can also be used to estimate the spike length of awned varieties. Anther sampling was carried out when the distance between the flag and boot leaf collars was 0–5 cm (the average distance being 2 cm) and when the spike length was 3–6 cm (mostly around 4–5 cm). A 0-5 cm collar interval and 3–6 cm spike length broadly corresponded to premeiotic interphase and meiosis within the oldest floret of the spike, but meiotic stage could not be differentiated further using external measurements. Spikes were sampled from the first (oldest) five tillers only. At mid-meiosis in Chinese Spring plants, there was a lag in development of around 3–4 days between the oldest spike and the spike from the 5th tiller.

### Anther sampling

To identify the meiotic stages immediately before and after temperature treatments, we used an anther sampling technique adapted from Bennett and Smith (1972) and Bennett et al. (1971, 1974). In a wheat spike, each floret contains three anthers, and the PMCs within any one of these three anthers are approximately synchronised in meiotic development with the PMCs of the other two anthers. This means that the meiotic stage of an anther removed pre-treatment can be compared with that of the two anthers from the same floret post-treatment, to determine whether high temperatures have had any effects on meiosis. Wheat has several florets per spikelet, but in these experiments, anthers were only sampled from either of the two most developed florets in the spikelet because these are almost always fertile under normal conditions.

**Fig. 1:**
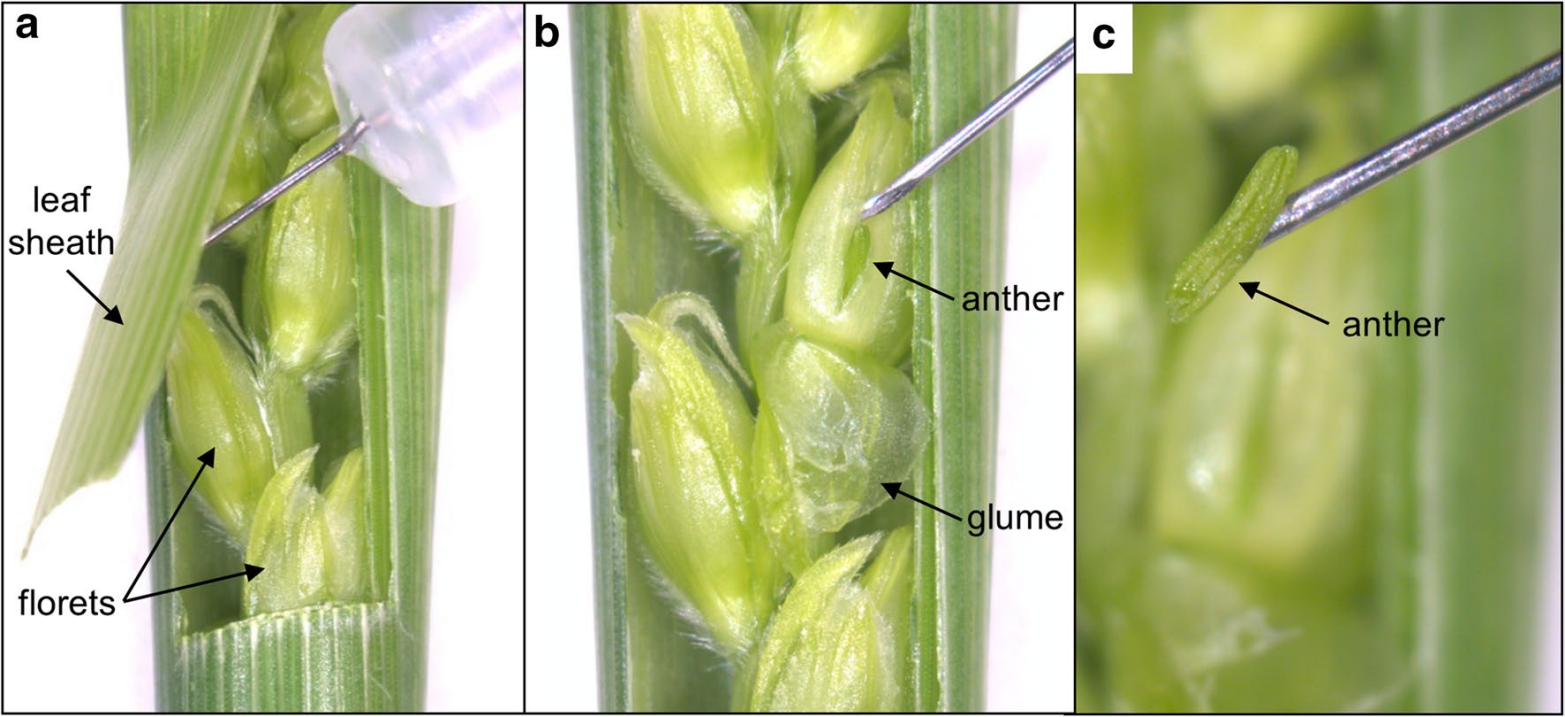
Anther sampling from an immature spike of a wheat plant at the ‘early booting’ stage of development; **a** dissection of a flap in the leaf sheaths to expose florets; **b** excision of a single anther through a slit in the oldest floret (note that glume has been folded back); **c** a single excised anther

Using an M80 stereo microscope (Leica Microsystems Ltd., Milton Keynes, UK) to aid dissection, a small flap of approximately 1.5 cm in length was carefully cut in the leaf sheath surrounding the developing spike to expose a small number of spikelets in the middle of the spike (Fig. 1a). There is a gradient of development within a wheat spike, with the oldest spikelet (i.e., most advanced in development) located mid-spike and progressively younger spikelets occurring towards the top and bottom of the spike. Anthers were always sampled from the floret at the most basal position of the oldest-looking spikelet to try to ensure that heat treatment was never applied to PMCs that were at a more advanced stage of development than those of the sampled anther. Once this floret had been identified, its glume was folded back and a slit was cut through the lemma to expose a single anther (Fig. 1b), which was then carefully excised intact (Fig. 1c). The anther was stained using acetocarmine, and squashed under a cover slip to extrude the PMCs. Using a DM2000 light microscope (Leica Microsystems), the PMCs were examined to determine the stage of premeiosis or meiosis. The two non-sampled anthers remained attached to the receptacle, and care was taken to avoid damage to these anthers and their delicate filaments. The sampled floret and spike remained attached to the plant. To minimise desiccation of the inflorescence, the flap was sealed immediately after sampling using a small, sterile adhesive dressing (sticking plaster). The absorbent pad of the dressing was soaked with water and placed against the wound site, and the sticky edges of the dressing were wrapped around the plant stem to seal the flap completely. The sampled tiller was enclosed in a polythene bag containing a few drops of water so that the excision site was covered. The plant was optimally watered then placed into a CER for a 20-h heat treatment.

In Chinese Spring, none of the distinguishable stages of premeiosis or meiosis last for more than 18 h at normal or higher temperatures; so it was reasonable to assume that a treatment period of 20 h would be long enough for the PMCs to have moved from one developmental stage to the next, to confirm meiotic progression, but short enough to enable us to determine which specific stages of meiosis are most affected by high temperatures. Meiotic timings are similar in euploid and N5DT5B plants; although in N5DT5B plants at 20 °C the total length for meiosis has been reported to be slightly longer than in euploids, lasting 25 h rather than 23.5 h (Bayliss and Riley 1972b).

### Temperature regimes

Following excision of the first anther, plants were exposed to higher than normal temperatures (30 or 35 °C) for 20 h, or were given a control treatment of 20 h at 20 °C. Treatments were given in a CER under continuous light and at approximately the same time of day for each treatment, starting in the early afternoon between 13:40 and 14:30 and finishing the following morning between 09:40 and 10:30. To prevent dehydration during treatment, humidity levels were maintained at 75%. Only one floret was sampled for each spike, which ensured that the size of the dissection opening was kept to a minimum to prevent excessive desiccation. To avoid damaging the remaining anthers within the floret, a great deal of care was needed to excise the first anther, which was time-consuming, so sampling only a single floret also limited the time of exposure of the immature spike to the open air.

After heat treatment, the two remaining anthers within the previously sampled floret were excised, and acetocarmine squashes were made as before. This allowed comparison of PMCs before and after exposure to high temperatures. Most of the time, the second and third anthers could be accessed via the original dissection site, but occasionally, the opening needed to be extended a little because the spike had grown and moved up within the leaf sheaths. The flap was sealed and the sampled tiller covered as before. The plant was returned to normal growing conditions in the original CER. Two days after the second anther excision, the bag was removed from the sampled tiller to reduce the likelihood of fungal infection occurring at the dissection site. By this time, the sampled spike had usually moved up within the leaf sheaths, away from the dissected flap, and desiccation of the spike via the dissection site was unlikely.

Heat treatments were given at different stages of development, ranging from premeiotic interphase through to the point at which young microspores have just been released from the tetrad walls and have yet to develop a germ pore (microspore release). PMCs were examined to determine whether meiosis (or premeiosis) progressed. For each temperature treatment, data from anther sampling of 11–19 individual plants was used in the analysis. For some plants, more than one spike was sampled, so data from 11 to 26 spikes per temperature treatment was used in the final analysis. For each excised anther, all PMCs were assessed rather than a selection of them. If the PMCs within a single anther were not completely synchronised in terms of meiotic stage (as was occasionally observed) the PMCs were scored according to the meiotic stage of the majority of cells. The relationship between temperature and the progression of PMCs through meiosis was assessed using Chi-square tests. Stacking bar charts were plotted using Microsoft Excel (2016).

### Grain number analysis

To provide controls for the grain number analysis, 8 Chinese Spring euploid plants and 12 N5DT5B plants were grown as above and exposed to 20 °C for 20 h without anther excision. The stage of floral development of the control plants was similar to that of the experimental plants at the time of each temperature treatment.

On ear emergence, the five oldest spikes on each experimental and control plant were enclosed within cellophane crossing bags (55 × 190 mm, Focus Packaging and Design Ltd, UK) to prevent cross-pollination. For the experimental plants, this normally included one dissected spike and four non-dissected spikes. At maturity, the total numbers of grains per bagged spike were counted. To investigate the relationship between temperature and grain number, data were analysed using a general linear model (*t* test), using Genstat 18th edition [VSN International (2015), Hemel Hempstead, UK]. Bar charts were plotted using SigmaPlot Version 13.0 (Systat Software Inc.).

### Meiotic staging

Assigning PMCs to the different stages of meiosis was carried out mainly with reference to Bennett et al. (1973). We used multiple criteria to stage the PMCs including size of nuclei, intensity of staining, whether individual chromosome threads were visible, the degree of contraction of the chromosomes, the number, size and position of the nucleoli and the presence or absence of callose on the PMC walls. The appearance of the tapetal cells (whether mono or binucleate and whether dividing) was noted, but was only used as a rough guide as this is a less reliable marker for staging meiosis.

During stage 1 of premeiotic interphase, PMC nuclei are poorly stained and have a diffuse net-like appearance, whereas during stage 2, the PMC nuclei become denser and more darkly staining. Stages 1 and 2 can be further distinguished by the fact that callose does not appear until stage 2. There are normally three nucleoli within each nucleus during both stages 1 and 2, but during stage 3, these usually reduce to form a single nucleolus, which migrates to the surface of the nucleus and can be seen to project from the surface. In Chinese Spring, after the end of stage 3, the tapetal cells undergo a highly synchronised mitotic cell division, giving rise to binucleate cells which persist throughout meiosis. The onset of tapetal cell divisions was originally used by Bennett et al. (1973) as a marker for the start of leptotene, but this is unreliable, because the association of a specific stage of PMC development with tapetal cell divisions varies with temperature (Bennett et al. 1979). Moreover, the tapetal cell divisions do not necessarily occur at leptotene in other hexaploid wheat varieties (Bennett and Smith 1972). At leptotene, the chromosomes begin to contract so that it is possible to start to distinguish chromosome threads, and at the start of zygotene there is a characteristic looping of chromosome threads at the periphery of the nucleus, as the chromosomes become more contracted. Stages from pachytene onwards were easier to distinguish and therefore are not described here.

### Verification of meiotic stages using FISH-labelled telomeres

The position and clustering status of telomeres during specific stages of premeiosis and early prophase was established using FISH (fluorescence in situ hybridisation) to ensure that scoring of the acetocarmine squashes was accurate in terms of meiotic stage, and to give an additional point of reference in terms of meiotic processes that are taking place. A pair of anthers was removed from each of eight florets from Chinese Spring euploid plants. For each floret sampled, the two outermost anthers were used in preference to the middle anther, because these were found to be more closely synchronised with each other in terms of meiotic stage. The first of the two anthers was stained with acetocarmine and squashed, and the second was prepared for FISH using the method of Cabrera et al. (2002). A telomere repeat sequence (Cox et al. 1993) was used to label the chromosomes in the FISH experiments, because the position and pairing status of wheat telomeres at the different stages of premeiosis and meiotic prophase has been previously characterised (Martinez-Perez et al. 1999). Chromosomes were counterstained with DAPI (4’, 6-diamidino-2-phenylindole, 1 μg/ml). Signals were visualised using a DM5500B fluorescence microscope (Leica Microsystems), and images were captured using a Hamamatsu Orca Flash 4.0 V2 scientific CMOS camera. Z-stacks were deconvolved using Leica LAS-X software and images were processed using Fiji image analysis software (Schindelin et al. 2012). It was not possible to FISH label all anthers in the heat experiments because it was too time-consuming.

## Results

### Assessment of the protocol: comparison of telomere dynamics with classical meiotic staging

Precise staging of the PMCs was a key part of this study, but defining the start of meiosis, and the transition points between the early stages of meiosis, using criteria visible by light microscopy is always somewhat subjective. However, comparison of PMCs from pairs of anthers from the same floret, either stained with acetocarmine or labelled with a telomeric repeat sequence (TRS), allowed us to link classical definitions of the meiotic stages with the position and clustering behaviour of telomeres during premeiosis and meiosis. This also provides a reference point for comparison of our study with other studies in the future.

**Fig. 2:**
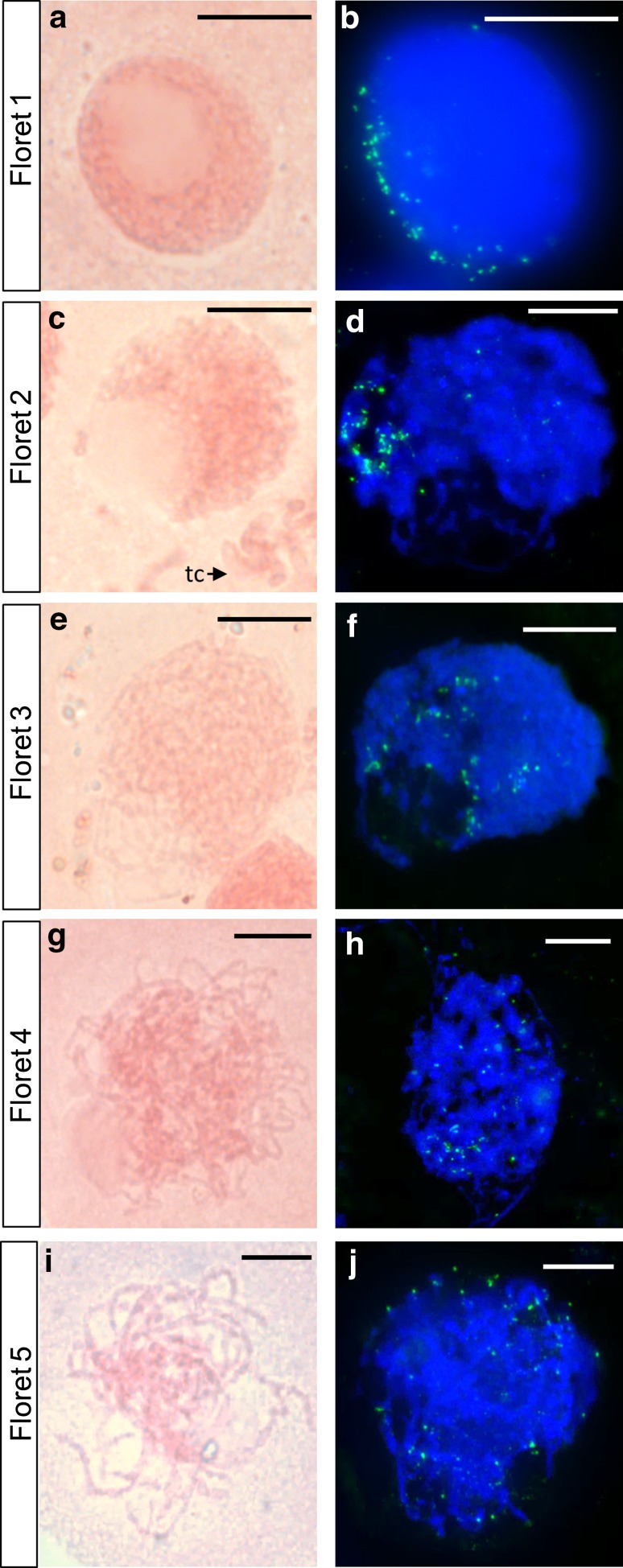
A comparison of Chinese Spring PMC nuclei from pairs of anthers taken from the same floret, squashed and stained with acetocarmine (**a**, **c**, **e**, **g**, **i**) or FISH labelled with a telomeric repeat sequence (*green*) and counterstained with DAPI (*blue*) (**b**, **d**, **f**, **h**, **j**): **a** and **b** premeiotic interphase stage 3/leptotene; **a** individual chromatin threads not visible; single nucleolus centrally situated in the nucleus; **b** telomeres distributed around the periphery of one hemisphere of the nucleus; **c** and **d** late leptotene; **c** nucleolus located at the periphery of the nucleus; a few chromosomes from a tapetal cell division are partially visible *bottom right* (tc); **d** telomeres loosely clustered at the periphery of the cell; **e** and **f** early zygotene; **e** individual chromosome threads are more easily discernible; **f** telomeres are more dispersed than in **d**; **g** and **h** late zygotene/early pachytene; **g** chromosomes more contracted than in **e** and individual chromatin threads more readily visible; **h** telomeres dispersed; **i** and **j** later pachytene; **i** chromatin thickening; **j** telomeres dispersed and present in a haploid number, indicative of pairing; *scale bar* 10 μm

Figure 2 shows PMCs from pairs of Chinese Spring euploid anthers from the same floret, the first anther having been stained with acetocarmine and squashed, and the second FISH labelled with TRS. In Fig. 2a the PMC nucleus from the first anther is densely stained and has no discernible individual chromatin threads. It has a single central nucleolus, although other PMCs in the same anther had a nucleolus at the periphery of the nucleus. Callose was clearly visible in this anther and there were no mitotic divisions in the tapetal cells. From the staging defined by Bennett et al. (1973), this would be classified as premeiotic interphase stage 3. In the second anther from the same floret, labelled with TRS, in most cells the telomeres were distributed around the periphery of one hemisphere of the nucleus as shown in Fig. 2b. Martinez-Perez et al. (1999) suggests that this configuration of telomeres occurs at late S-phase/onset of meiotic prophase, so this means that the acetocarmine scoring and the FISH scoring assign both anthers to the same stage.

However, a small proportion of PMCs within the second anther had telomeres that were tightly clustered at the periphery of the nucleus. In many organisms, the telomeres form a cluster or ‘bouquet’ on the inner surface of the nuclear envelope in early prophase (Scherthan et al. 1996; Bass et al. 1997). Using classical definitions of meiotic stages such as those of Bennett et al. (1973), in hexaploid wheat, telomeres would begin to cluster during late premeiotic interphase, *before* the stage classically defined as leptotene. However, more recent studies using FISH-labelled chromosomes have shown that, when telomeres cluster, the labelled chromosomes do not have a typical premeiotic interphase configuration, and it has been concluded that the telomere bouquet stage in wheat occurs at the onset of leptotene (Martinez-Perez et al. 1999). So, although most PMCs within this anther are at late premeiotic interphase, a small proportion of PMCs must already be at leptotene. This is not particularly surprising because in our temperature treatment experiments we occasionally observed a small degree of asynchrony within a single anther in terms of meiotic stage. Each anther has four loculi, or pollen sacs, containing the PMCs. Asynchrony sometimes occurred within a single loculus, where a slight developmental gradient could be seen between PMCs within the pollen sac, and sometimes it occurred between loculi within the same anther.

In Fig. 2c the nucleolus is located at the periphery of the PMC nucleus and the chromatin appears less tightly packed than in the premeiotic interphase nucleus shown in Fig. 2a. This PMC was scored as late leptotene. Tapetal cells were undergoing mitosis at this stage, and a few of the mitotic chromosomes are partially visible in Fig. 2c. In the FISH-labelled anther from the same floret, the telomeres are loosely clustered at the periphery of the cell (Fig. 2d), which indicates that PMCs in this anther too are at leptotene. In Fig. 2e the acetocarmine stained PMC is at early zygotene. Chromatin has become thread-like, and characteristic looping of chromosome threads can be observed around the periphery of the nucleus, particularly around the nucleolus, which can be seen at the bottom left of the nucleus. In the corresponding FISH-labelled PMCs, in some nuclei the telomeres were still loosely clustered, often co-localising with the nucleolus, whilst in others the telomere cluster appears to have started to disperse (Fig. 2f). Telomere clustering is normally maintained in wheat until the end of zygotene (Martinez-Perez et al. 1999).

Figure 2g shows a PMC nucleus at late zygotene/early pachytene. The chromosome threads are more contracted, appearing thicker and more easily distinguishable than in the nucleus in Fig. 2e. In the equivalent FISH-labelled anther (Fig. 2h), the telomeres have dispersed across the whole nucleus, although there are still more sites in the lower hemisphere than in the upper. Figure 2i shows a late pachytene nucleus, and in the equivalent FISH-labelled anther the telomeres have fully dispersed but tend to be distributed towards the periphery of the nuclei (Fig. 2j); it is possible to count a haploid number of telomeres indicating that the telomeres are paired. In some nuclei, the telomere pairs could be seen as two adjacent dots.

### Effects of high temperatures on meiotic progression

**Fig. 3:**
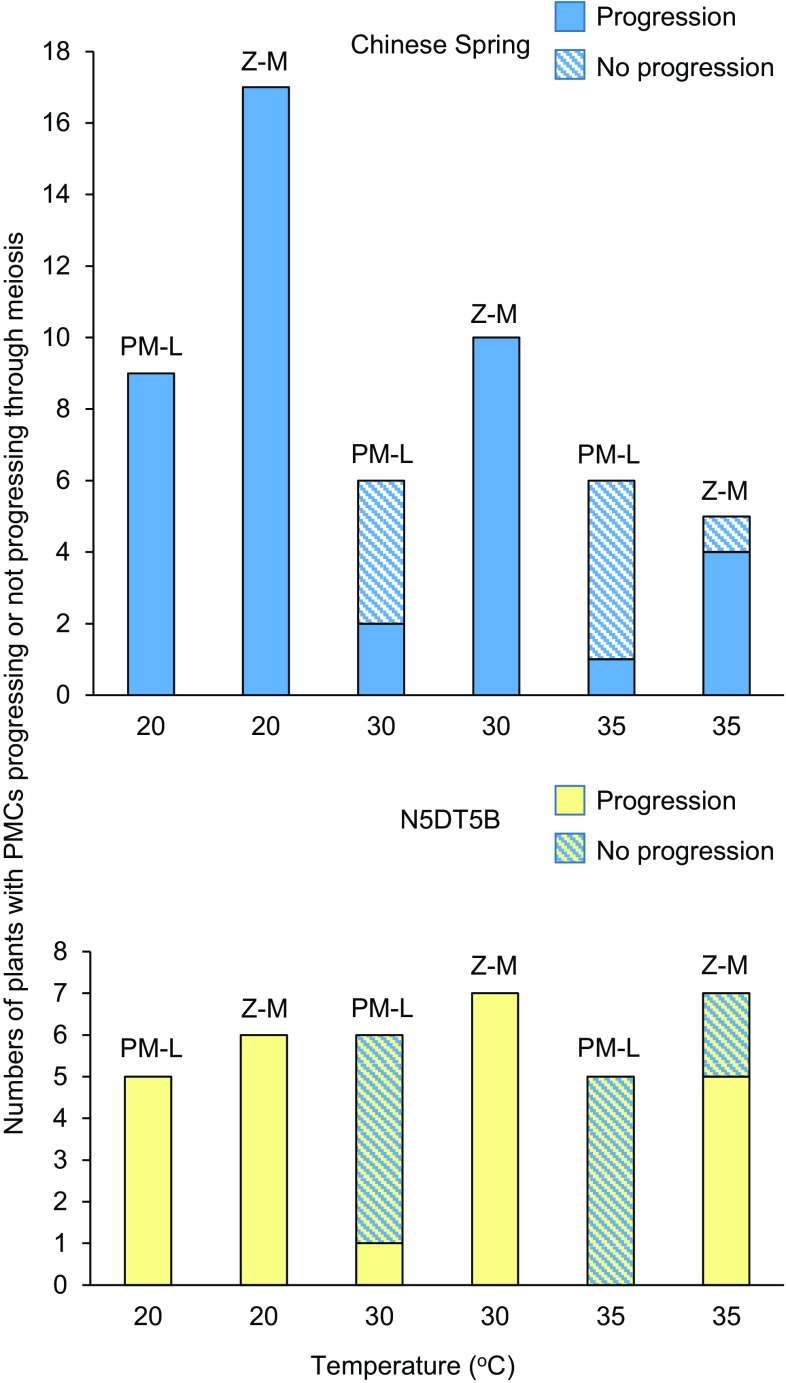
Stacked *bar chart* showing numbers of Chinese Spring euploid plants (*blue bars*) or N5DT5B plants (*yellow bars*) with PMCs progressing (*solid bars*) or not progressing (*patterned bars*) through meiosis after exposure to 20, 30 or 35 °C during either premeiosis–leptotene (PM-L) or zygotene–microspore (Z-M)

Our data show that exposure of wheat to higher than normal temperatures affects the progression of PMCs through meiosis (Table 1; Fig. 3). Progression was considered to have taken place if PMCs in the anthers sampled post-treatment were at a later stage than PMCs in anthers from the same floret sampled pre-treatment. For analysis of meiotic progression, we divided the stages of floral development into two groups: (1) premeiotic interphase to late leptotene (up to the leptotene–zygotene transition) and (2) zygotene to young microspore as defined above.

**Table 1:** Numbers of Chinese Spring euploid or N5DT5B plants with pollen mother cells progressing or not progressing through meiosis after exposure to different temperatures at two different stages of floral development

Temperature	Pre-treatment stage	Chinese spring euploid		N5DT5B	
		Meiotic progression	No progression	Meiotic progression	No progression
20 °C	Premeiosis–leptotene	9	0	5	0
	Zygotene–microspore	17	0	6	0
30 °C	Premeiosis–leptotene	2	4	1	5
	Zygotene–microspore	10	0	7	0
35 °C	Premeiosis–leptotene	1	5	0	5
	Zygotene–microspore	4	1	5	2

**Fig. 4:**
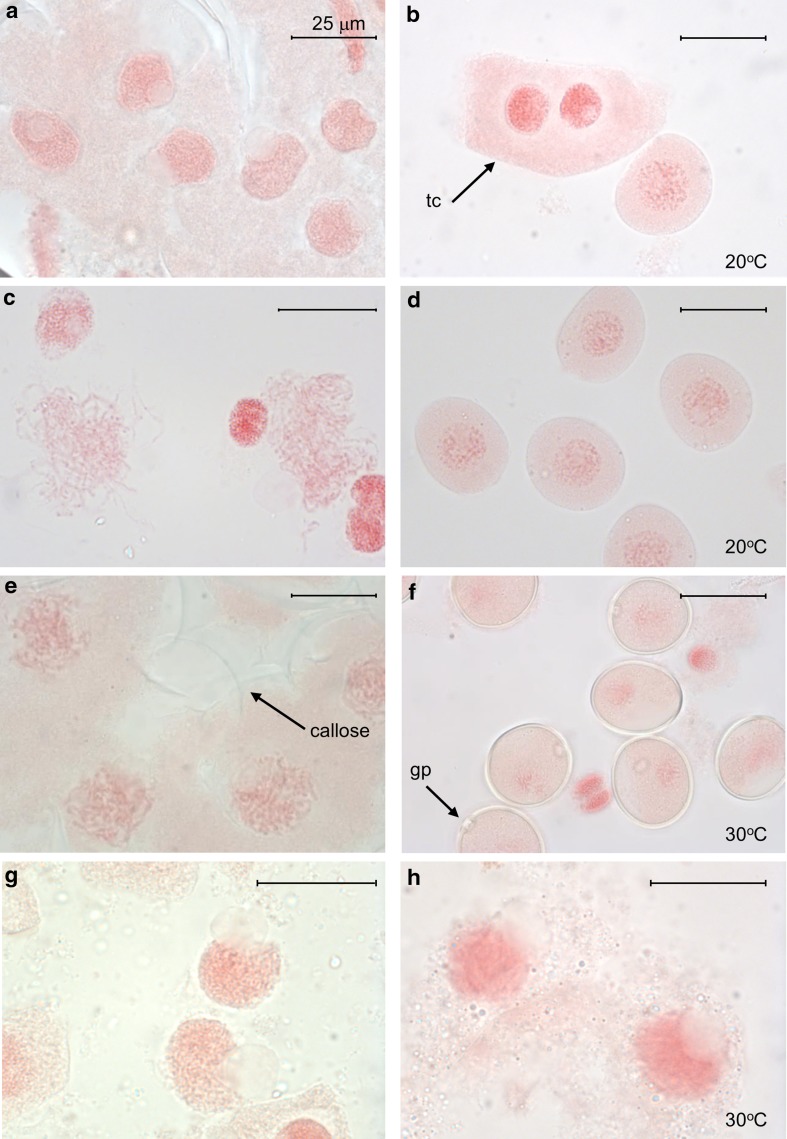
Nuclei of PMCs from Chinese Spring euploid anthers squashed and stained with acetocarmine: **a** nuclei at leptotene before heat treatment; nuclei are densely staining; a single nucleolus projects from the surface of most nuclei; **b** nuclei from an anther from the same floret sampled after 20 h at 20 °C; PMCs have progressed to the early microspore stage; a binucleate tapetal cell (tc) is shown for comparison; **c** nuclei at late zygotene before heat treatment; **d** nuclei from the same floret sampled after 20 h at 20 °C; PMCs have progressed to the early microspore stage; **e** nuclei at zygotene before heat treatment; callose (labelled with an *arrow*) is visible; **f** nuclei from the same floret after 20 h at 30 °C; PMCs have progressed to the microspore stage; a germ pore (gp) is visible in most cells; **g** nuclei at leptotene before heat treatment; **h** nuclei from the same floret sampled after heat treatment at 30 °C; PMCs have not progressed through meiosis, and nuclei appear to be degrading. *Scale bar*, 25 μm

When Chinese Spring euploid or N5DT5B plants were exposed to 20 °C for 20 h (normal temperatures), all PMCs progressed normally through meiosis, as expected, irrespective of the developmental stage prior to the treatment period. Figure 4a–d shows Chinese Spring euploid PMCs from before and after treatment at 20 °C to illustrate normal progression through meiosis at this temperature.

When plants were exposed to 30 or 35 °C, however, the PMCs showed a difference in response dependent on the stage at which the plant was exposed. All Chinese Spring and N5DT5B PMCs exposed to 30 °C during the zygotene–microspore stages progressed normally through meiosis. Figures 4e and f show an example of normal progression in Chinese Spring at 30 °C. However, if plants were exposed to the 30 °C treatment during any period from premeiotic interphase to late leptotene, only 2 out of 6 Chinese Spring euploid plants and 1 out of 6 N5DT5B plants had PMCs that progressed, which was significantly fewer than expected (*P* < 0.01). Where there was no progression (Fig. 4g, h), in a small proportion of the anthers, some of the heat-treated cells had an appearance which might indicate degradation (Fig. 4h). This was also seen in some anthers at 35 °C.

Exposure at 35 °C had an even greater effect on progression than exposure at 30 °C. At this temperature, progression was affected in plants treated at the zygotene–microspore stages as well as those treated at premeiosis–leptotene. For plants treated at 35 °C during the zygotene–microspore stages, 4 out of 5 Chinese Spring euploid plants and 5 out of 7 N5DT5B plants progressed through meiosis. However, the most pronounced effect was seen on plants exposed to 35 °C at premeiosis–leptotene. At this temperature, progression through meiosis was seen in only 1 out of 6 Chinese Spring plants and none of the 5 N5DT5B plants studied, which was, again, significantly fewer than expected (*P* < 0.01). Although a slightly higher proportion of Chinese Spring euploid plants had PMCs that progressed through meiosis at both 30 and 35 °C, we did not identify a significant difference in progression between Chinese Spring euploid and N5DT5B PMCs (*P* = 0.4, Chi-square test).

### Effects of high temperatures on grain number per spike

**Fig. 5:**
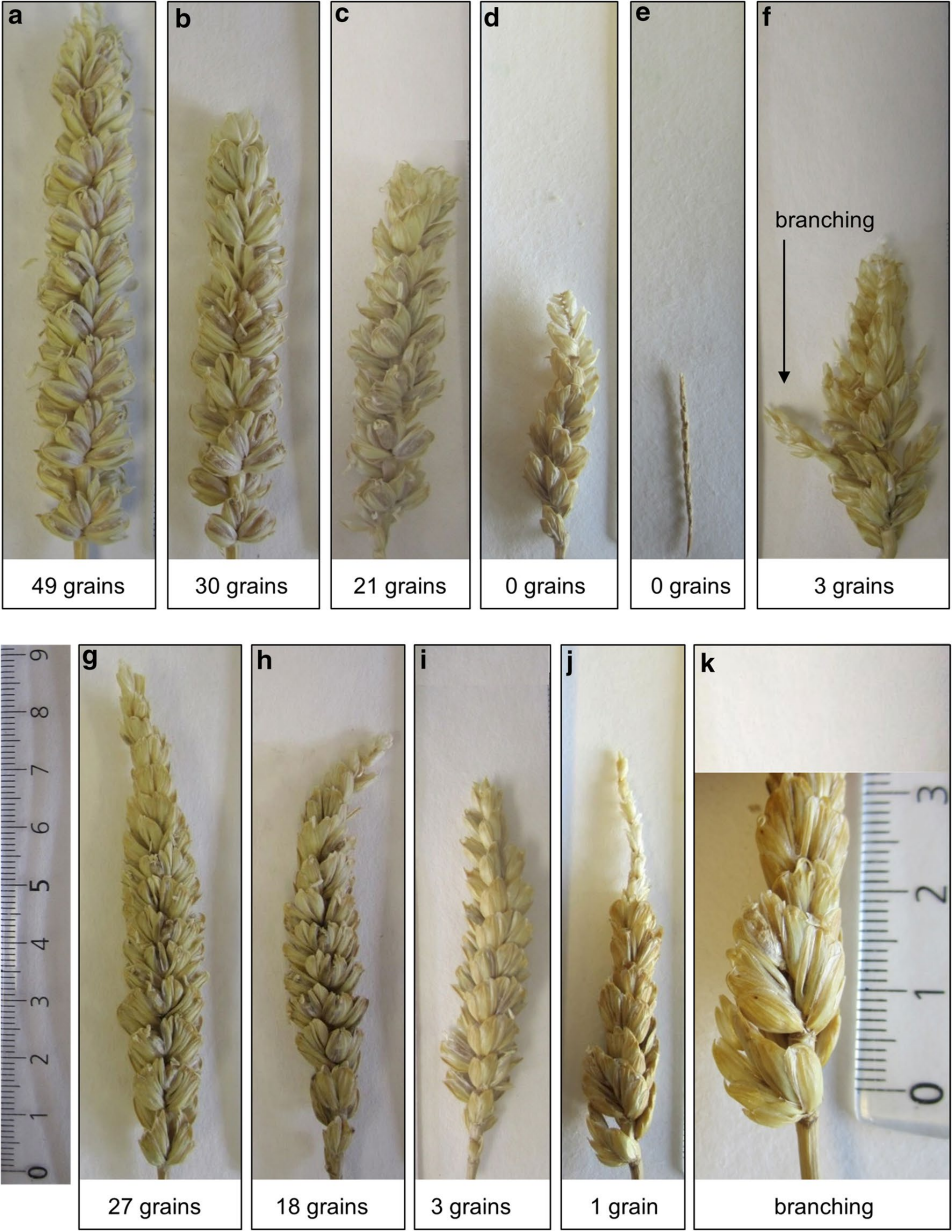
Typical examples of mature Chinese Spring spikes after exposure to different temperatures for short periods during floral development; note reduction in spike length and grain number as temperature increases; **a**–**f** Chinese Spring euploid spikes exposed to different temperatures for 20 h (**a**–**e**) or 24 h (**f)**; **a** non-dissected spike after exposure to 20 °C; **b**–**f** anther sampled spikes after exposure to 20 °C (**b**), 30 °C (**c**) or 35 °C (**d**, **e** and **f**); **e** spike did not emerge; development arrested at time of temperature treatment; **f** branching visible; **g**–**k** N5DT5B spikes after exposure to different temperatures; **g** non-dissected spike exposed to 20 °C; note tapering ear shape and reduced fertility in upper part of spike; **h**, **i** and **j** anther sampled spikes after exposure to 20 °C (**h**), 30 °C (**i**) and 35 °C (**j**); **k** close-up of lower part of dissected spike shown in **j**; branching visible in lower spikelets; *scale* in cm

**Fig. 6:**
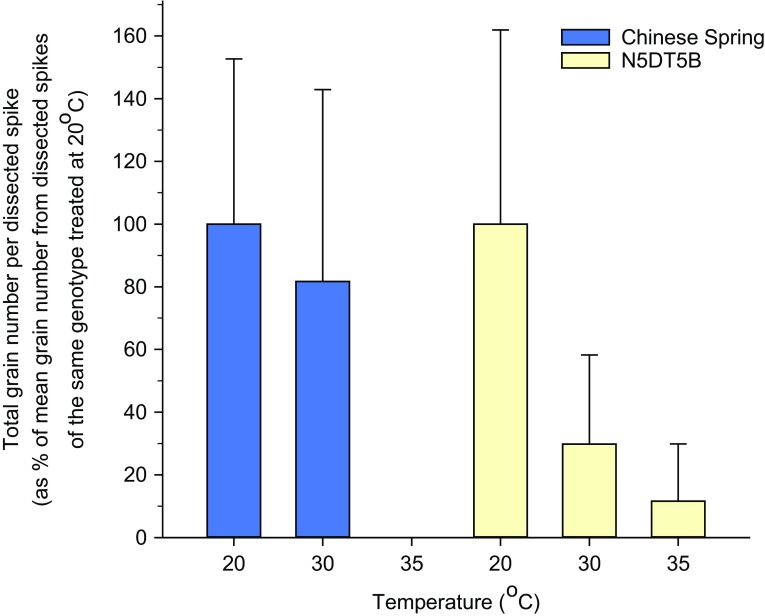
*Bar chart* showing total grain number per dissected spike of Chinese Spring euploid plants (*blue bars*) or N5DT5B plants (*yellow bars*) after exposure to 20, 30 or 35 °C for 20 h during premeiotic interphase or meiosis. Grain number per dissected spike is expressed as a percentage of mean grain number from all dissected spikes of the same genotype treated at 20 °C. *Error bars* show standard deviation

Following treatment at 20, 30 or 35 °C, total grain number per spike was counted in the five oldest spikes of each plant. In the control group of plants with no dissected anthers, after treatment at 20 °C, the mean total grain number per spike produced by Chinese Spring plants was 54, which was double the mean of 27 for the N5DT5B plants. Similar results were obtained from non-dissected spikes of plants with a dissected spike: Following treatment at 20 °C, the mean total grain number per non-dissected spike from Chinese Spring euploid plants was 50, which was close to double the mean of 28 for the N5DT5B spikes. Figures 5a, g show typical examples of these spikes. Clearly, at 20 °C, N5DT5B plants have an inherently lower fertility than Chinese Spring euploid plants. Therefore, to make it easier to compare the grain number results from the two different genotypes, we standardised the data by expressing grain number per dissected spike as a percentage of the mean number of grains from all dissected spikes of the same genotype treated at 20 °C. The results are shown in Table 2 and Fig. 6.

**Table 2:** Grain number per dissected spike of Chinese Spring euploid and N5DT5B plants after exposure to different temperatures during floral development

Genotype	Chinese spring euploid			N5DT5B		
Temperature	20 °C	30 °C	35 °C	20 °C	30 °C	35 °C
Grain number per dissected spike (%)^a^	40.9	61.4	0.0	139.4	15.5	7.7
	40.9	0.0	0.0	139.4	23.2	31.0
	30.7	0.0	0.0	69.7	77.5	7.7
	54.6	88.7	0.0	240.1	15.5	62.0
	136.5	30.7	0.0	108.4	93.0	15.5
	47.8	129.6	0.0	108.4	15.5	7.7
	119.4	160.4	0.0	23.2	54.2	7.7
	139.9	71.6	0.0	38.7	15.5	0.0
	102.4	156.9	0.0	46.5	0.0	0.0
	112.6	34.1	0.0	123.9	15.5	0.0
	139.9	75.1	0.0	62.0	31.0	0.0
	13.6	0.0			0.0	0.0
	0.0	105.8			31.0	
	68.2	54.6				
	122.8	174.0				
	143.3	163.8				
	112.6					
	153.5					
	133.1					
	88.7					
	197.9					
	153.5					
	92.1					
	133.1					
	44.4					
	177.4					
Mean	100.0	81.7	0.0*	100.0	29.8*	11.6*
SD	52.7	61.2	0.0	61.9	28.0	18.2

* *P* < 0.001 (*t* test)

^a^ Grain number per dissected spike (%) = total grain number per dissected spike expressed as a percentage of mean grain number from all dissected spikes of the same genotype following exposure to a control temperature of 20 °C

Within the Chinese Spring euploid 20 °C treatment group, 26 spikes were dissected: 17 spikes with the oldest anther staged at zygotene to microspore and only 9 spikes where the oldest anther was staged from premeiosis–leptotene. In all other treatment groups, there was a more even balance between older and younger spikes. To make the data more balanced, we randomly selected 9 of the 17 euploid spikes from the 20 °C group scored at zygotene–microspore to use in the data analysis, leaving 8 of the older spikes out of the analysis. Thus, data analysis was carried out using 9 premeiosis–leptotene and 9 zygotene–microspore spikes. Data analysis was repeated 10 times using a different set of 9 randomly selected plants each time. The results and statistical significances of the data analysis for the 10 sub-sets of spikes were all very similar to those obtained from analysing data from all 26 spikes within this treatment group; so we have only described the results and statistical significances of the whole group, and it is the results for the whole group that are shown in Table 2 and Fig. 6.

Figure 5 shows that in both Chinese Spring euploid and N5DT5B plants there was a reduction in both spike length and grain number as temperature increased. In the euploid plants, there was a reduction in mean grain number from 100% at 20 °C to 82% after treatment at 30 °C, which was not a statistically significant reduction, but following the 35 °C treatment, Chinese Spring euploid plants failed to produce any grain (Figs. 5d, e), so the exposure of plants to this temperature had a highly significant effect (*P* < 0.001) on grain number (Table 2; Fig. 6). However, it should be noted that we have observed the occasional euploid plant producing around three or four grains when exposed to 35 °C for a 24-h period (example in Fig. 5f).

For the N5DT5B plants, mean grain number per dissected spike dropped from 100% at 20 °C to 30% at 30 °C and 12% at 35 °C. The reductions in grain number were highly significant (*P* < 0.001) at *both* temperatures. The same significant results were obtained if non-standardised data was used in the analysis. At 35 °C we also noted that in both genotypes sometimes spike development was arrested, presumably at the time of heat treatment/dissection, and that the spike did not emerge (Fig. 5e). At this temperature, we occasionally observed branching in the spike (Fig. 5f, j, k). This branching was not observed at the lower temperatures.

### Does heat arrest meiotic progression temporarily or permanently?

An experiment was carried out to determine whether meiosis completely aborts after heat treatment or whether it temporarily stalls until the temperature returns to normal levels. This was undertaken by repeating the anther sampling experiments described above, sampling a first anther pre-heat treatment and a second anther from the same floret post-treatment. The plant was then allowed a recovery period of 24 h under normal temperature and light conditions before the remaining (third) anther from the sampled floret was excised and fixed. Unfortunately, in almost all cases, none of the third anthers had scorable PMCs. The cellular material had become rather amorphous in these anthers and it was difficult to identify the PMCs. The third anther often appeared slightly less turgid than normal, indicative of water loss.

## Discussion

### Accuracy and practicality in the staging of meiosis and premeiosis

Accurate scoring of the stages of meiosis and premeiosis was important in this study, but we needed to score a large number of anthers, so our protocol could not be too labour-intensive. Staging using the anther sampling method provided more precision than using morphological characteristics visible to the naked eye, but it was too time-consuming to stage all the PMCs using more precise methods for labelling chromatin such as FISH.

We identified an occasional lack of consensus in the scientific literature between scoring of meiotic stages using traditional staining methods and scoring using fluorescent labelling techniques. Martinez-Perez et al. (1999) established that, in wheat, the telomere bouquet appears at the onset of leptotene, but other authors suggest that the bouquet stage occurs later, at the leptotene–zygotene transition (Zickler and Kleckner 1998), and that this is a universal phenomenon. Part of the issue may be connected with using the appearance of the nucleolus for meiotic staging. For example, Bass et al. (1997), working on maize, describe the nucleolus as being large, single and *centrally located* at leptotene, whereas Bennett et al. (1973), working on wheat, state that the nucleolus has already migrated to the surface of the nucleus by the end of premeiotic stage 3. It is unclear whether this difference in interpretation of stage in terms of nucleolus position is due to differences in meiosis between maize and wheat or lack of consensus in scoring.

Our own labelling experiments revealed that the telomeres were distributed around the periphery of one hemisphere of the nucleus during stage 3 of premeiotic interphase (Fig. 2b), and were clustered at leptotene, indicating that the telomere bouquet stage had been reached (Fig. 2d). These stages correspond to the temperature-sensitive period as defined in our experiments. The position and clustering status of the telomeres provide a useful guide because they tell us what is happening to the chromosomes during early meiosis. During the telomere bouquet stage, chromosomes align and synapse from their telomere regions. The paired telomeres then disperse and chromosome synapsis is completed. The comparison of acetocarmine stained PMCs with those with labelled telomeres gave us more confidence in the accuracy of our scoring of anthers in the temperature experiments.

We occasionally observed a small degree of asynchrony between the three anthers within a single floret, more commonly between a side anther and middle anther than between the two side anthers (the outermost anthers). Other studies have also found that meiotic synchrony does not always occur between anthers in the same floret (Richards et al. 2012). This asynchrony could partially explain why some of the plants scored as being at a particular developmental stage had PMCs that progressed through meiosis after heat treatment, and some of them had PMCs that showed no progression despite being scored as being at the same stage (Table 1).

Anther excision by dissection was necessary to score the meiotic stages with precision. A comparison of different anther sampling methods involving risk of damage to anthers or no risk has previously shown that damage caused by dissection is insignificant (Bennett et al. 1971), but other studies have suggested that anther excision can increase the sensitivity of anther development to high temperature (Bennett et al. 1972). Our data showed an apparent reduction in grain number in the dissected spikes when compared with grain number in the non-dissected spikes at 20 °C (Fig. 5, data not shown), suggesting that anther excision does have an effect, but this could not be accurately assessed because without anther excision in the non-dissected spikes it is not possible to ascertain the meiotic stage at which they are exposed to the different temperature treatments.

In our meiotic progression experiments, all sampled spikes were treated the same in terms of dissection, so any dissection effect should be similar for all plants, and, in fact, when plants were exposed to normal temperatures (20 °C) after anther sampling, all PMCs progressed normally through meiosis irrespective of developmental stage. However, younger anthers could potentially be more susceptible to dehydration at higher temperatures. We observed that, in the early stages of premeiosis, the anthers within a floret are bathed in fluid, which gradually disappears as meiosis progresses. It is possible that this fluid affords some protection against high temperature and that anther excision may compromise it in some way that might affect anther development.

### Temperature sensitivity during premeiosis and early meiosis

We have demonstrated that exposure of wheat plants to less than 24 h of high temperature during premeiosis–leptotene can prevent normal progression of PMCs through the meiotic cell cycle. Bayliss and Riley (1972b) also identified a temperature-sensitive stage in wheat during premeiotic interphase, which subsequently affected chromosome pairing and chiasma formation; however, in their case, the scored PMCs clearly progressed, suggesting that we are identifying a different sensitive period during this stage.

In our study, most PMCs progressed through meiosis if they had reached zygotene by the time heat was applied. Thus, it seems likely that that there are processes that are critically affected by high temperatures during premeiotic interphase–leptotene. During the latter stages of premeiotic interphase, DNA replication takes place, while during early meiosis, leptotene chromosomes undergo complex changes in chromatin organisation, and there are dynamic movements of the telomeres as they form the telomere bouquet. During late leptotene each chromosome finds its homologue and the homologous chromosomes begin to associate in pairs. Correct pairing of the homologous chromosomes is vital for maintaining the stability and fertility of the genome.

### The effects of heat on grain number

High temperatures also led to a reduction in grain number in both Chinese Spring euploid and N5DT5B plants, with the reduction being greatest at the highest temperature. The reduction in grain number was highly significant at 35 °C for both genotypes and also highly significant at 30 °C for N5DT5B plants. In euploid plants exposed to 30 °C, the reduction in grain number from 100% (at 20 °C) to 82% was not significant, probably due to the large variation that generally occurred in grain number between spikes even within the same temperature treatment groups. It was not possible to correlate reduced grain number with heat sensitivity at a specific meiotic stage, because there can be differences in development of up to three days between different florets within a single wheat spike (Saini and Aspinall 1982).

The results of the grain number experiments suggest that in PMCs that have not progressed, it is more likely that meiosis has aborted completely rather than arrested temporarily, but it is difficult to draw any firm conclusions because high temperatures can affect other cells and organs that play a part in wheat fertility. For example, the female gametes can also be affected by heat stress during meiosis resulting in a reduction in seed set (Saini et al. 1983), and high temperatures can cause tapetal cell wall thickening, which has also been associated with male sterility (Bennett et al. 1972). Interestingly, spike length was also reduced as temperatures increased. We cannot infer that reduced spike length is a consequence of arrested meiosis because both meiotic and non-meiotic processes can be affected by heat, but it is possible that meiotic arrest could result in some sort of feedback mechanism whereby problems with grain set affect the growth of the other parts of the spike.

### Chromosome 5D stabilises the wheat genome at high temperatures

When chromosome 5D is absent, the proportion of PMCs progressing through meiosis after treating at 30 and 35 °C at the sensitive stage is slightly lower than when 5D is present. In fact, for PMCs exposed to 35 °C at this stage, where plants lack chromosome 5D there is no progression at all. However, we could not detect a significant difference in meiotic progression between the two genotypes, possibly because the experiment was not large enough.

There is, however, a difference in temperature sensitivity between the two genotypes in terms of grain number at 30 °C. Following exposure to 35 °C, Chinese Spring plants produced virtually no seed, and, although N5DT5B plants had a slightly higher grain number, the reduction in grain number was highly significant for both genotypes at this temperature. Following treatment at 30 °C, however, there was a highly significant reduction in grain number in the N5DT5B plants, whereas the reduction in grain number in Chinese Spring euploid plants was not significant. In fact, grain number was reduced by more than two-thirds in plants lacking chromosome 5D compared to when 5D was present. This marked reduction in grain number is unlikely to be the result of progression issues because the progression of PMCs was not affected by treatment of 30 °C during the zygotene–microspore stage and there was only a slight difference between the progression of Chinese Spring euploid and N5DT5B PMCs after a treatment of 30 °C at premeiosis–leptotene. This greater heat sensitivity of N5DT5B plants supports the conclusions of Bayliss and Riley (1972a) that the presence of a gene or genes on chromosome 5D stabilises the wheat genome against the effects of high temperatures during meiosis.

Heat tolerance is thought to be a complex trait, likely to be under the control of multiple genes (Barnabás et al. 2008). Two heat shock genes, *Hsp20* and *Hsp90* (Griffiths et al. 2006), have been identified on the long arms of the group 5 chromosomes, and it has been postulated that there are genes on chromosomes 5DL (*Ltp1*), 5DS (*Ltr*), 5BS (*Ltp3*) and 5AS (*Ltp2*) that have a stabilising effect at low temperatures (Queiroz et al. 1991), but these latter genes have not been mapped. In general, little is known about genes controlling tolerance of high temperatures in wheat.

### Does high temperature arrest meiotic progression temporarily or permanently?

It is not known whether meiosis completely aborts after heat treatment or whether it temporarily stalls until the temperature returns to normal levels. To address this question, a third anther was sampled following a recovery period at 20 °C, but the PMCs were not scoreable. It was relatively easy to extrude the first (pre-heat treatment) anther through a small slit in the lemma, but to excise the second anther it was necessary to cut open and peel back the lemma to expose the two remaining anthers. It is likely that the loss of turgidity and inability to score PMCs in the third anther is a result of desiccation or damage to this anther during the excision of the second anther. The presence of PMCs that appear to be degenerating after heat treatment suggests that, at least in these cells, meiosis would be permanently arrested. However, this degradation was only seen in a small proportion of PMCs where there was no progression.

The reduced meiotic progression of PMCs as temperatures reach 30 °C and higher, may be a mechanism by which the plant temporarily delays meiosis or premeiosis, preventing PMCs from progressing through meiosis when temperatures are too high during the hottest parts of the day. Delays in meiotic progression of 8–25 h have been previously demonstrated in *Arabidopsis thaliana* meiotic mutants (Higgins et al. 2004; Jackson et al. 2006). It has been suggested that plants lack robust checkpoints that result in a failure to complete meiosis, but instead may possess a surveillance mechanism that senses problems during meiosis and delays progression (Jones and Franklin 2007). However, in maize and rice *am1* mutants, meiosis arrests prematurely during leptotene, leading the authors to suggest the presence of a meiotic checkpoint at leptotene/zygotene (Che et al. 2011; Pawlowski et al. 2009). Moreover, Zheng et al. (2014) suggest that in Arabidopsis, the cyclin dependent kinase CDKG1 could be part of a checkpoint mechanism that detects recombination defects and leads to meiotic arrest. Interestingly, CDKG1 is essential for the completion of chromosome synapsis at high ambient temperature.

### Implications for plant breeding

The most temperature-sensitive stages of meiosis are normally over before the highest temperatures occur later in the day. When day temperatures increase above a certain threshold level, it may be that some wheat varieties delay meiosis so that the most sensitive stages are not exposed to high temperatures. When the temperature reduces (for example during evening and early morning), meiotic progression may continue. If meiotic progression can be delayed by high temperatures, current screening for heat tolerant wheat may simply select for wheat whose meiosis occurs earlier in the morning, rather than for wheat varieties whose meiosis is fundamentally more heat tolerant. This could be a problem if night temperatures increase beyond a certain threshold level, because the plant will no longer be able to escape the high temperatures simply by delaying meiosis. It is therefore important to identify wheat varieties in which the early meiotic stages are inherently more heat tolerant rather than those with an ability to delay meiosis to escape high temperatures. If true thermotolerance genes can be identified, new wheat cultivars can be developed that will be better adapted to future temperature increases. However, in our experience, anther sampling is time-consuming and it is not an approach that could be easily used to screen for temperature tolerant wheat.

#### Author contribution statement

TD carried out all practical work including experimental design, analysed the data, produced the figures and wrote the manuscript. GM provided the concept, provided thoughts and guidance and revised and edited the manuscript.
